# Age related differences in cannabis use and subjective effects in a large population-based survey of adult athletes

**DOI:** 10.1186/s42238-019-0006-9

**Published:** 2019-07-29

**Authors:** Joanna S. Zeiger, William S. Silvers, Edward M. Fleegler, Robert S. Zeiger

**Affiliations:** 1 Canna Research Group, 3996 Savannah Ct., Boulder, CO 80301 USA; 2 Division of Allergy and Clinical Immunology, University of Colorado Denver School of Medicine, 12700 E. 19th Ave., Room 10C03, Aurora, CO 80045 USA; 3 To-Life in Peace, LLC, 3812 Taft Court, Wheat Ridge, Colorado 80033 USA; 4 Kaiser Permanente Southern California, 7060 Clairemont Mesa Blvd, San Diego, CA 92111 USA

**Keywords:** Marijuana, Medical marijuana, Adults, Sports medicine

## Abstract

**Background:**

There is a paucity of information regarding cannabis use behaviors in adult community-based athletes as most research in athletes has focused on misuse of cannabis in elite, adolescent, university-based athletes. We aimed to determine whether age related differences exist in patterns of cannabis use and subjective effects to cannabis in adult athletes.

**Methods:**

The Athlete PEACE Survey used mainly social media and email blasts to recruit and SurveyGizmo to collect data. Cannabis patterns of use (duration of use, frequency of use, routes of administration, cannabinoid used, concurrent use with exercise), benefits, and adverse effects were reported. Age was reported by decade from 21 to ≥60. Age trends in cannabis use patterns and subjective effects were assessed using linear trend analysis.

**Results:**

Of the 1161 participants, 301 (26%) athletes currently used cannabis. Younger athletes compared to older athletes reported significantly more positive and adverse subjective effects to cannabis, used cannabis longer, and used both tetrahydrocannabinol and cannabidiol for medical and recreational purposes. Younger athletes used cannabis concurrently with exercise more often than older athletes and consumed edibles, vaporized, and smoked more than older athletes.

**Conclusions:**

We found age-related cannabis patterns of use and subjective effects to cannabis. Concerns about cannabis mis-use and abuse in athletes maybe overstated with the potential benefits (improved sleep, decreased anxiety, less pain) outweighing the adverse effects (increased anxiety, increased appetite, difficulty concentrating).

**Electronic supplementary material:**

The online version of this article (10.1186/s42238-019-0006-9) contains supplementary material, which is available to authorized users.

## Background

Athletes are known as early adopters of new and innovative modalities to aid in recovery and performance (Conrad et al. 2019; Caine et al. 2012), particularly since pain, insomnia, and anxiety are common and difficult to solve problems among athletes (Hainline et al. 2017; Mann et al. 2007; Halson 2014). Cannabis is a modality that has reportedly improved symptoms among sufferers of pain, insomnia, and anxiety (Stith et al. 2019; Mannucci et al. 2017), but has rarely been studied in the context of adult athlete use. Historically, cannabis use in athletes has typically been studied in the framework of abuse among adolescent, university, and elite athletes (Ware et al. 2018; Buckman et al. 2011; Peretti-Watel et al. 2003a). More recent studies have suggested that athletes are using cannabis to improve mood and enjoyment of exercise (YorkWilliams et al. 2019), but patterns of use and positive and adverse effects to cannabis in adult athletes is largely unknown.

The NCAA found that 30.3% of student athletes consumed cannabis with social and recreational reasons as the primary reasons for use (Brisola-Santos et al. 2016). International studies reported that current cannabis use ranged from 2.7 to 23.0% in elite athletes (Brisola-Santos et al. 2016). Studies in elite and collegiate athletes found the highest use in males, winter sport athletes, and teenage females who compete on an international level (Brisola-Santos et al. 2016). These studies did not measure self-reported effects to cannabis.

It is critical to unravel the self-reported effects to cannabis (subjective effects) as cannabis use increases due to legalization and consumption for medical purposes. Measuring subjective effects and relating them to patterns of use, cannabinoids used, routes of administration, and age-related differences can provide insights for athletes, consumers, and medical professionals to develop best practices for using cannabis to treat medical conditions. Subjective effects have been collected to understand many facets of cannabis use and abuse (Haberstick et al. 2011; Scherrer et al. 2009). In adolescents, cannabis subjective effects are a predictor of downstream use and abuse (Haberstick et al. 2011; Zeiger et al. 2010), while in laboratory settings subjective effects are used to understand the dosing effects of tetrahydrocannabinol (THC) (Curran and Brignell 2002; Schwope et al. 2012). A randomized-controlled clinical trial in Australian cannabis users and non-users aged 21–44, evaluated CBD alone, THC alone, THC combined with high CBD, THC combined with low CBD, and a placebo (Solowij et al. 2019). The study concluded that low doses of CBD in combination with THC enhanced the effects of THC while high doses of CBD in combination with THC reduced the effects of THC (Solowij et al. 2019), indicating that the dosing effects of cannabinoids changed the perceived effects of cannabis use. Longitudinal studies of subjective effects to cannabis have shown that initial positive and adverse responses to cannabis in adolescence predict cannabis use behavior in young adulthood.

A review of subjective effects to cannabis found that relaxation was the most commonly reported item (Green et al. 2003), but paradoxical impacts of cannabis can occur in which opposing effects can be experienced within individuals and between individuals (Green et al. 2003; Zeiger et al. 2012). That individuals can experience both positive and adverse effects to cannabis may partially be due to the lack of information on optimal dosing and/or the possible inverted U-shaped dose-response curve on efficacy at low doses and adverse effects at high doses (Solowij et al. 2019; Martin-Santos et al. 2012; Zuardi et al. 2017).

Age-effects for past month cannabis use exist; the highest use was observed in 18–25-year old’s (17.3%) and the lowest use among those over 50 (2.0%) (Haug et al. 2017)\. Younger cannabis users showed a higher prevalence of cannabis use disorders and middle-aged and older adults reporting using cannabis medically (Haug et al. 2017). A cross-sectional study of age-related patterns of cannabis use in dispensary patients found that 75% were males, the frequency of cannabis use did not differ across ages but the amount consumed differed with the younger cannabis users (18–21) consuming a larger quantity of cannabis than middle-aged users (31–50) and older users (51–74). Routes of administration differed by age group, with younger cannabis users showing a preference for vaping, older age groups using oral administration, and the oldest age group reporting medical cannabis use more frequently than the younger ages (Haug et al. 2017).

Patterns of use are related to subjective effects to cannabis (Daniulaityte et al. 2018; Ware et al. 2005). Frequency of use, route of administration, and type of cannabinoid used all impact the effects to cannabis (Stith et al. 2019; Sexton et al. 2016; Stith et al. 2018). A comprehensive study of medical cannabis patients found that the primary route of administration was inhalation (84%); oral administration was only 8, and 0.6% used topicals (Peretti-Watel et al. 2003b).

Age-related subjective effects to cannabis and patterns of cannabis use in adults are poorly elucidated particularly in adult community-based athletes. As cannabis becomes legal in more states, cannabis use will continue to rise, both for medical and recreational purposes. It is important to understand the age-related differences in cannabis use among adult athletes, particularly for those athletes who want to receive the most benefit with lowest risk, for physicians who manage these athletes, and for health policy makers who develop guidelines for cannabis use among athletes and the general population.

Our Athlete Pain, Exercise, And Cannabis Experience (PEACE) Survey study presently in review (Buckman et al. 2011) characterized cannabis use using cluster analysis in community-based athletes ≥21 years of age. The objectives of the present secondary analysis of the Athlete PEACE Survey study were to determine in adult athletes (1) whether age-related differences occur in patterns of cannabis use (i.e. frequency and duration of use, route of administration, reason for use, primary cannabinoid used, timing around exercise) exist and (2) whether positive and adverse subjective effects to cannabis differ by age. We hypothesized that there would be age trends in cannabis patterns of use and subjective effects to cannabis.

## Methods

This cross-sectional quantitative survey study used a convenience sample (Zeiger et al. 2019a). The study was approved with waiver of written consent by Solutions IRB (http://www.solutionsirb.com). Participants were assured confidentiality. Implied consent was provided by survey completion. Participants were required to be, (1) ages 21 years or older, (2) a self-declared athlete of any sport, and (3) English speaking with no other inclusions or exclusions. The survey was administered on SurveyGizmo (https://www.surveygizmo.com) between 6 September 2018 and 7 December 2018. Social media (Facebook, Twitter, LinkedIn, athlete forums), email communications, and flyers posted in specialty sports stores in Boulder and Baltimore were used for subject recruitment, allowing for large scale targeting of potential participants in a relatively short time (Zeiger and Zeiger 2018). 1161 (91.1%) of the 1274 athletes taking the survey completed it (see Additional file 1 for survey questions).

Demographics were collected and reported in Additional file 2: Table S1 (Zeiger et al. 2019b). Athletes were asked whether they ever used marijuana and “In the past two weeks, have you used marijuana [including THC and/or cannabidiol (CBD)]?” Participants who responded “yes” to using marijuana in the past 2 weeks were asked if they primarily used THC, CBD, or both THC and CBD. Questions about positive (Table 2, 9 items) and adverse (Table 3, 8 items) subjective effects from cannabis use were included. Participants were able to endorse as many of the items that applied to them. Routes of administration were also measured; participants were able to choose as many as they used. This was a secondary analysis of a primary cluster analysis study. Sample size justifications for cluster analysis are the following: A systematic analysis of sample sizes for cluster analyses reviewed 243 cluster analyses. The study found that the median sample size for the cluster analyses was 293 participants, similar to the 301 participants used in the cluster analysis of the primary study (Online and Dolnicar 2002). A simulation study found valid solutions for cluster analysis with samples as small as 20 (Henry et al. 2015). Our results from the primary analysis manuscript showed that the present sample size was adequate to clearly cluster the participants into 3 clinically distinct clusters (Zeiger et al. 2019a).

Descriptive analyses were conducted using SPSS v23. Three hundred one of the 1161 (25.9%) survey completers were current cannabis users and are the subject for this secondary analysis which examines the age-related differences of cannabis use. The Jonckheere-Terpstra procedure, a non-parametric rank based trend test in SPSS, was performed to determine whether there was a statistically significant linear trend between the ordinal variable of age by decade and the patterns of use and subjective effects (Bewick et al. 2004). The null hypothesis of the study states that there will be no age-related differences in cannabis patterns of use and subjective effects to cannabis as measured by the Jonckheer-Terpstra procedure. *P* values < 0.05, 2 sided, was set for significance.

## Results

Of current cannabis users (*n* = 301), 15.6% were 21–29 (*n* = 47), 24.6% were 30–39 (*n* = 74), 26.9% were 40–49 (*n* = 81), 20.3% were 50–59 (*n* = 61) and 12.6% were ≥ 60 years of age (*n* = 38). The sample was 60.3% male and 89.1% Caucasian with no difference in age distribution by gender or ethnicity (Additional file 2: Table S1). Demographics, sports demographics, attitudes about cannabis and patterns of cannabis use by primary sport are shown in Additional file 2: Table S1. The “other” sports category was comprised of swimming, winter sports, hiking, walking, climbing, yoga, trail running, and strength sports. The “other” sport category reported the most frequent cannabis use, but there were no differences by sport for reasons for cannabis use, duration of cannabis use, and cannabinoid used. Triathletes exercised the greatest number of days per week for the most hours per week and more frequently reported being “professional” or a serious/competitive amateur. Among current cannabis users, 61.1% indicated that they use cannabis for pain with no significant difference in use for pain by sport. Athletes in the “other” sports category (36.5%) reported using cannabis within 1 h of exercising more often than the other sports; there were no differences for cannabis use during exercise or within 1 h after exercise by sport (Additional file 3: Table S2).

### Patterns of use (Table 1)

Younger athletes used both THC and CBD (*p* = 0.009) and older athletes used mainly CBD only (*p* < 0.001). A significant trend was seen for reason for use with younger athletes using cannabis more for recreational (*p* = 0.002) and both THC and CBD for medical and recreational reasons (p = 0.009), while older athletes used cannabis for medical purposes (p < 0.001). A longer duration of use trended towards younger athletes (p < 0.001), but frequency of use did not differ by age. Younger athletes consumed edibles, smoked, and vaporized more often than older athletes (all *p* < 0.001); older athletes used oil/tinctures at a higher rate than younger athletes (*p* = 0.003). Overall, 26.2% of the sample reported cannabis use within 1 h before starting exercise, 9% indicated cannabis use during exercise, and 33.2% indicated they use cannabis within 1 h after exercise. There were significant age trends for cannabis use prior to exercise and after exercise with younger athletes using cannabis more often during those time periods than older athletes (p < 0.001). Athletes were asked whether they used cannabis within 1 h before exercise, during exercise, and within 1 h after exercise. Cannabis use during exercise was not common, ranging from 4.3% in athletes 21–29 to 11.1% in athletes 40–49; there was not a significant trend for age for use during exercise. Athletes primarily used cannabis within one of hour of exercise for improving focus (46.3%) and improving activity enjoyment (47.8%). Athletes used cannabis within one-hour post-exercise to aid in recovery (75.4%), for pain management (67.9%) and for sleep enhancement (65.7%) (Additional file 3: Table S2).

**Table 1:** Cannabis patterns of use by decade in community-based athletes

Pattern of use	Category	21 to 29 (*n* = 47)	30 to 39 (*n* = 74)	40 to 49 (*n* = 81)	50 to 59 (*n* = 61)	≥60 (*n* = 38)	*P* value for trend
	N (%)						
Cannabinoid used	THC	10 (21.3)	19 (25.7)	16 (19.8)	10 (16.4)	6 (15.8)	0.21
	CBD	9 (19.1)	16 (21.6)	27 (33.3)	28 (45.9)	21 (55.3)	< 0.001
	THC and CBD	28 (59.6)	39 (52.7)	38 (46.9)	23 (37.7)	11 (28.9)	0.009
Reason for use	Medical	4 (8.5)	15 (20.3)	28 (34.6)	31 (50.8)	21 (55.3)	< 0.001
	Recreational	21 (44.7)	23 (31.1)	23 (28.4)	15 (24.6)	5 (13.2)	0.002
	Both	22 (46.8)	36 (48.6)	30 (37.0)	15 (24.6)	12 (31.6)	0.004
Duration	> 3 years	30 (63.8)	41 (55.4)	37 (45.7)	21 (34.4)	18 (47.4)	< 0.001
Frequency	≥4 times/week	12 (25.5)	39 (52.7)	33 (40.7)	20 (32.8)	13 (34.2)	0.20
Routes of administration	Edible	31 (66.0)	38 (51.4)	46 (56.8)	18 (29.5)	14 (36.8)	< 0.001
	Vaporize	25 (53.2)	34 (45.9)	28 (34.6)	14 (23.0)	8 (21.1)	< 0.001
	Smoke	23 (48.9)	35 (47.3)	26 (32.1)	15 (24.6)	8 (21.1)	< 0.001
	Oil/tincture	17 (36.2)	24 (32.4)	35 (43.2)	31 (50.8)	23 (60.5)	0.003
	Topical	15 (31.9)	23 (31.1)	29 (35.8)	22 (36.1)	13 (34.2)	0.57
	Capsule	7 (14.9)	8 (10.8)	13 (16.0)	10 (16.4)	1 (2.6)	0.51
	Spray	2 (4.3)	5 (6.8)	3 (3.7)	3 (4.9)	1 (2.6)	0.57
Routes of administration	Edible	31 (66.0)	38 (51.4)	46 (56.8)	18 (29.5)	14 (36.8)	< 0.001
	Vaporize	25 (53.2)	34 (45.9)	28 (34.6)	14 (23.0)	8 (21.1)	< 0.001
	Smoke	23 (48.9)	35 (47.3)	26 (32.1)	15 (24.6)	8 (21.1)	< 0.001
	Oil/tincture	17 (36.2)	24 (32.4)	35 (43.2)	31 (50.8)	23 (60.5)	0.003
	Topical	15 (31.9)	23 (31.1)	29 (35.8)	22 (36.1)	13 (34.2)	0.57
	Capsule	7 (14.9)	8 (10.8)	13 (16.0)	10 (16.4)	1 (2.6)	0.51
	Spray	2 (4.3)	5 (6.8)	3 (3.7)	3 (4.9)	1 (2.6)	0.57
Timing of use	Within 1 h before starting exercise	14 (29.8)	25 (33.8)	21 (25.)	13 (21.3)	6 (15.8)	< 0.001
	During exercise	2 (4.3)	7 (9.5)	9 (11.1)	6 (9.8)	3 (7.9)	0.53
	Within 1 h after finishing exercise	21 (44.7)	37 (50.0)	21 (25.9)	13 (21.3)	8 (21.1)	< 0.001

### Positive subjective effects (Table 2)

Six of the nine positive subjective effect items from cannabis use showed a significant trend towards younger athletes. These positive effects included help with sleep, calm, decreased anxiety, euphoria, decreased nausea, and increased energy. Less pain, fewer muscle spasms and improved athletic performance did not differ by age strata.

**Table 2:** Endorsement of positive subjective effects to cannabis by decade in community-based athletes

Positive subject effects	21 to 29 (*N* = 47)	30 to 39 (*N* = 74)	40 to 49 (*N* = 81)	50 to 59 (*N* = 61)	≥60 (*n* = 38)	P value for trend
	N (%)					
Helps with sleep	37 (78.7)	61 (82.4)	59 (72.8)	35 (57.4)	23 (60.5)	0.003
Calms me down	36 (76.6)	59 (79.7)	37 (45.7)	26 (42.6)	18 (47.4)	< 0.001
Less pain	32 (68.1)	54 (73.0)	56 (69.1)	37 (60.7)	28 (73.7)	0.48
Decreased anxiety	30 (63.8)	52 (70.3)	44 (54.3)	25 (41.0)	13 (34.2)	< 0.001
Euphoria	20 (42.6)	31 (41.9)	16 (19.8)	16 (26.2)	6 (15.8)	< 0.001
Decreased nausea	17 (36.2)	22 (29.7)	11 (13.6)	7 (11.5)	3 (7.9)	< 0.001
Increased energy	12 (25.5)	29 (39.2)	21 (25.9)	14 (23.0)	4 (10.5)	0.019
Fewer muscle spasms	10 (21.3)	16 (21.6)	16 (19.8)	4 (6.6)	4 (10.5)	0.063
Improved athletic performance	9 (19.1)	18 (24.3)	15 (18.5)	8 (13.1)	6 (15.8)	0.18

### Adverse subjective effects (Table 3)

Adverse subjective effects were endorsed far less frequently than positive subjective effects; however, differences were noted by age strata. Significant trends for adverse effects were observed with higher endorsement for younger than older athletes for anxiety, increased appetite, cardiovascular (e.g. increased heart rate, palpitations), respiratory (e.g. wheezing, coughing, itchy eyes), gastrointestinal (e.g. vomiting, diarrhea, nausea), and poorer athletic performance.

**Table 3:** Endorsement of adverse subjective effects to cannabis by decade in community-based athletes

Negative subject effects	21 to 29 (*N* = 47)	30 to 39 (*N* = 74)	40 to 49 (*N* = 81)	50 to 59 (*N* = 61)	≥60 (*n* = 38)	P value for trend
	N (%)					
Anxiety	21 (44.7)	23 (31.1)	11 (13.6)	7 (11.5)	1 (2.6)	< 0.001
Increased appetite	18 (38.3)	25 (33.8)	12 (14.8)	10 (16.4)	8 (21.1)	0.002
Difficulty concentrating	14 (29.8)	12 (16.2)	10 (12.3)	8 (13.1)	6 (15.8)	0.063
Cardiovascular	9 (19.1)	8 (10.8)	1 (1.2)	3 (4.9)	0 (0.0)	< 0.001
Respiratory	8 (17.0)	19 (25.7)	6 (7.4)	8 (13.1)	3 (7.9)	0.002
Gastrointestinal	5 (10.6)	4 (5.4)	1 (1.2)	0 (0.0)	0 (0.0)	0.001
Worse athletic performance	1 (2.1)	7 (9.5)	1 (1.2)	0 (0.0)	0 (0.0)	0.003
Skin reaction	0 (0.0)	1 (1.4)	2 (2.5)	0 (0.0)	0 (0.0)	0.81

## Discussion

The present secondary analysis of a larger study (Zeiger et al. 2019a) examined patterns of cannabis use and subjective effects to cannabis by decade in adult community-based athletes. Significant differences were observed across decades for cannabis use patterns and reported subjective effects to cannabis. Younger athletes significantly used cannabis longer, consumed more THC only and in combination with CBD for recreational and combined medical/recreational purposes than older athletes. Younger athletes endorsed more positive and adverse subjective effects items than older athletes.

The different patterns of use and subjective effects to cannabis by age group in adults are novel findings since few studies have looked at specific age related effects to cannabis. A very small study reported that young adults (*n* = 20, 24–28 years) reported more acute subjective effects (anxiety and less alertness) of inhaled cannabis than 20 adolescents (16–17 years) (Mokrysz et al. 2016). A cross-sectional study of adult users showed that older adults endorsed fewer positive and adverse subjective effects than younger adults, however older adults and those who used cannabis medically experienced the most acute withdrawal effects (Sexton et al. 2019). The difference in subjective effects to cannabis by age may stem from younger athletes consuming THC and THC/CBD combination more often than older athletes. An analysis of these data that explored the differences in subjective effects to cannabis by cannabinoid type suggested that athletes endorsed more positive and negative subjective effects to THC/CBD combination use (Zeiger et al. 2019c). The age related trends of subjective effects to cannabis may point to a biological difference in response to cannabis exposure in younger athletes or a willingness to use higher doses of THC via edibles or smoking, both of which can lead to a higher preponderance of positive and adverse effects which can sometimes lead to overdose (Meacham et al. 2018; Schauer et al. 2016; Monte et al. 2019). A study of medical cannabis users in California who were aged 18–72 found that younger users reported the highest quantity of use while older participants reported the fewest negative consequences related to cannabis use (Haug et al. 2017). Cohort effects of social acceptability, legal consequences, and legalization all may play a role in cannabis initiation, route of administration, and adoption of medical vs. recreation cannabis use (Haug et al. 2017). Although studies examining brain morphology have been disparate in their results, there is evidence that chronic cannabis users experience changes in brain structure and function, however it is unknown whether these changes are responsible for difference in subjective effects to cannabis by age (Lorenzetti et al. 2010; Batalla et al. 2013). Further studies need to be conducted in a larger replication sample and with clinical trials to better parse out the combined effects of THC and CBD and compare subjective and objective reactions to cannabis use by age. Reassuring of the clinical relevance of subjective effects, a recent trial found a moderate but statistically significant correlation between observed and self-reported subjective effects of intoxication between THC-only and low dose CBD in combination with THC (Solowij et al. 2019).

The use of cannabis to treat pain has been of interest. Less pain associated with cannabis use was reported from about 60 to 70% of athletes in all the age strata in the present study, highlighting that the reported positive effect of cannabis on reducing pain appears independent of age. The 69% frequency of pain relief in the present study across all ages is on the higher end of benefit reported in other studies noting an efficacy ranging from 37 to 86% for pain relief from cannabis (Ware et al. 2005; Brunt et al. 2014; Swift et al. 2005; Whiting et al. 2015).

Adverse effects were reported in this sample; however, at a lower reported rate than positive effects. Respiratory and cardiovascular adverse effects and anxiety showed the highest reporting in the younger age strata. This is possibly due to the route of administration since there was a tendency for the younger ages in this sample to smoke, vaporize, and consume edibles, while older age groups reported mostly using topicals, tinctures, and oils. Additionally, route of administration has been associated with subjective effects to cannabis use (Russell et al. 2018) and the preferred cannabinoids could also play a role in the reporting of adverse (and positive) effects (Niesink and van Laar 2013).

Our findings suggest that adult athletes are using cannabis both similarly and differently than adolescent and university athletes as well as the general population. Twenty-six percent of our overall sample reported current cannabis use, a frequency that is similar to that found in the NCAA, but lower than the overall population past-months use of 8.1% (Chawla et al. 2018). Past month use in a national sample showed lower frequencies of cannabis use across all ages than our sample of adult athletes (Chawla et al. 2018); national sample numbers ranged from 15.9% in 22–25 years old’s to 2.0% for those over 50. In our athlete sample, 33.3% of 21 to 29-year old’s consumed cannabis in the last 2 weeks, and 22.0 and 19.2% of those 50 to 59 and 60 and older used cannabis in the past 2 weeks, respectively.

The older adult athletes reported using cannabis for medical purposes with a preference for oral routes of administration, findings similar to other studies (Haug et al. 2017). Over 30% of this athlete sample reported using topical administration, a rate that is higher than reported in other studies (0.6–5%) (Russell et al. 2018). The frequency of 32.9% medical-only cannabis use was higher in this sample than the 17% reported in the National Survey on Drug Use and Health (Green et al. 2003). Cross-use of medical and recreational cannabis was observed in 38.2% of our athletes, which is lower than the 55% of medical and recreational combination use seen in a survey of 348 medical cannabis users (Zeiger et al. 2012). Furthermore, medical patients use cannabis more frequently than our athlete population, with 75% reporting using cannabis more than once per day in published studies (Zuardi et al. 2017). Studies of cannabis use indicate that past-months use is higher in males than females (Chawla et al. 2018). Our sample skewed male (62.3%), however, there were no significant differences by sex for current use.

It is challenging to put the results of the present study into the context of use in adolescent, university-based, and elite athletes. University athletes are mostly younger than the legal age for cannabis use and elite and university athletes are subjected to drug testing. However, drug testing among athletes is complicated by the fact that cannabis is a threshold drug, meaning athletes can use it out of competition up to a certain detectable blood THC level and THC is entirely banned within competition (Ware et al. 2018). The testing standards among elite athletes are different than occupational drug testing where any presence of cannabis may be grounds for discipline. Indeed, in our sample 20.5% of those who do not currently use cannabis reported they do not use it because it is not legal at their job. These complexities justify the importance of studying adult athletes apart from the traditional groups of adolescent, university, and elites.

Cannabis use concurrently with exercise was lower in this sample than the 81.7% found in a previous report, and unlike that study we did not find a difference by sex (data not shown); however, we did corroborate the finding that younger adults tended to use cannabis before and after exercise more often than older adults (YorkWilliams et al. 2019). In the previous study, the main reasons for cannabis use concurrent with exercise were enhanced performance, enjoyment, motivation, and recovery. In our study, we captured reasons for concurrent cannabis use with exercise separately for pre- and post- exercise; athletes in our study used cannabis for different reasons before exercise (improve focus, enjoyment) than after exercise (pain management, relaxation, aid in sleep). Our sample of athletes is unique in that 86.7% of participants indicated they exercise more than 5 h per week (45.9% reported ≥11 h per week), a number that exceeds the 2.66 h per week of the concurrent cannabis users in the YorkWilliams study and the daily WHO recommendation of 1 h per day (Kahlmeier et al. 2015). Concerns about cannabis mis-use and abuse in athletes maybe overstated with the potential benefits (improved sleep, decreased anxiety, less pain) outweighing the adverse effects (increased anxiety, increased appetite, difficulty concentrating).

Several limitations exist. Questionnaire based studies are always subject to misclassification errors. The internal consistency of the responses lends credibility to the participants answers. A moderate but statistically significant correlation between observed and self-reported subjective effects of intoxication between THC and low dose CBD in combination with THC in a recent trial, results which are encouraging for self-reported data collection. (Solowij et al. 2019). The study did not ask about dosing or ratios of CBD and THC in those who used both CBD and THC which limits the specificity of this combination. The generalizability of this convenience sample drawn from social media outlets is unknown, particularly due to the over-representation of Caucasians. In addition, it is not known whether the age effects observed on cannabis use in athletes will be seen in non-athletes. The study of cannabis users was of moderate size, but confirmation in larger cohorts would be informative. These analyses are exploratory and do not correct for multiple testing; the results, however will inform future analyses with data reduction techniques that will enable multivariate analysis.

The strength of this study is the online recruitment of a relatively large sample in a short amount of time. Community-based adult athletes have been infrequently studied with respect to cannabis use, patterns of use, or subjective effects to cannabis (Campian et al. 2018). Most studies of cannabis use in athletes have been mainly in adolescents, university athletes, and elite athletes (Ware et al. 2018; Buckman et al. 2011; Peretti-Watel et al. 2003a).

These analyses suggest there are age related differences in cannabis patterns of use and subjective effects to cannabis. The younger athletes compared to older athletes in this cohort reported more positive and adverse effects to cannabis, used cannabis longer, and consumed both CBD and THC for medical and recreational purposes. Future directions include examining age by cannabinoid type interactions in relation to subjective effects to cannabis.

## Additional files

Additional file 1: Study questionnaire. (DOCX 21 kb)

Additional file 2: **Table S1.** Demographics and cannabis patterns of use by primary sport. (DOCX 27 kb)

Additional file 3: **Table S2.** Endorsement of reason for use within one hour before, during, and within one hour after exercise by age group. (DOCX 19 kb)

## Data Availability

The datasets used and/or analyzed during the current study are available from the corresponding author on reasonable request.

## Abbreviations

CBD: Cannabidiol
THC: Tetrahydrocannabinol
The Athlete PEACE Survey: The Athlete Pain, Exercise, and Cannabis Experience Survey
