# Supplementary material for: Age related differences in cannabis use and subjective effects in a large population-based survey of adult athletes
Source: J Cannabis Res. 2019 Jul 29;1:7. doi: 10.1186/s42238-019-0006-9 (PMC7819305; doi:10.1186/s42238-019-0006-9)
Supplement: Supplementary file 1 — Study questionnaire. (DOCX 21 kb) [file 42238_2019_6_MOESM1_ESM.docx]

**Supplementary Table 1: Demographics and cannabis patterns of use by primary sport**

| Feature | Pattern of use | Primary Sport (n=301) | | | | | | | | | | |
| --- | --- | --- | --- | --- | --- | --- | --- | --- | --- | --- | --- | --- |
|  |  | Running  (n=75) | | Cycling  (n=69) | | Triathlon  (n=72) | | Other*  (n=85) | | Total  (n=301) | |  |
|  |  | n | % | n | % | n | % | n | % | n | % |  |
| Sex‡ | Male | 39 | 52.0 | 54 | 78.3 | 47 | 65.3 | 41 | 48.2 | 181 | 60.1 |  |
|  | Female | 36 | 48.0 | 15 | 21.7 | 25 | 34.7 | 44 | 51.8 | 120 | 39.9 |  |
| Ethnicity | White | 67 | 89.3 | 58 | 84.1 | 67 | 93.1 | 76 | 89.4 | 268 | 89.0 |  |
|  | Other | 8 | 10.7 | 11 | 15.9 | 5 | 6.9 | 9 | 10.6 | 33 | 11.0 |  |
| Age‡ | 21 to 29 | 10 | 13.3 | 4 | 5.8 | 13 | 18.1 | 20 | 23.5 | 47 | 15.6 |  |
|  | 30 to 39 | 22 | 29.3 | 8 | 11.6 | 17 | 23.6 | 27 | 31.8 | 74 | 24.6 |  |
|  | 40 to 49 | 29 | 38.7 | 23 | 33.3 | 15 | 20.8 | 14 | 16.5 | 81 | 26.9 |  |
|  | 50 to 59 | 11 | 14.7 | 17 | 24.6 | 21 | 29.2 | 12 | 14.1 | 61 | 20.3 |  |
|  | 60 and older | 3 | 4.0 | 17 | 24.6 | 6 | 8.3 | 12 | 14.1 | 38 | 12.6 |  |
| Type of athlete‡ | Professional | 2 | 2.7 | 0 | 0.0 | 8 | 11.1 | 1 | 1.2 | 11 | 3.7 |  |
|  | Serious/competitive athlete (amateur) | 19 | 25.3 | 19 | 27.5 | 45 | 62.5 | 16 | 18.8 | 99 | 32.9 |  |
|  | Frequent/fitness athlete | 31 | 41.3 | 30 | 43.5 | 14 | 19.4 | 25 | 29.4 | 100 | 33.2 |  |
|  | Recreational athlete | 23 | 30.7 | 19 | 27.5 | 5 | 6.9 | 39 | 45.9 | 86 | 28.6 |  |
|  | Other | 0 | 0.0 | 1 | 1.4 | 0 | 0.0 | 4 | 4.7 | 5 | 1.7 |  |
| Days/week exercise‡ | 1 | 1 | 1.3 | 0 | 0.0 | 0 | 0.0 | 5 | 5.9 | 6 | 2.0 |  |
|  | 2 | 1 | 1.3 | 2 | 2.9 | 0 | 0.0 | 5 | 5.9 | 8 | 2.7 |  |
|  | 3 | 5 | 6.7 | 11 | 15.9 | 5 | 6.9 | 15 | 17.6 | 36 | 12.0 |  |
|  | 4 | 18 | 24.0 | 20 | 29.0 | 4 | 5.6 | 20 | 23.5 | 62 | 20.6 |  |
|  | 5 | 18 | 24.0 | 17 | 24.6 | 8 | 11.1 | 16 | 18.8 | 59 | 19.6 |  |
|  | 6 | 19 | 25.3 | 14 | 20.3 | 29 | 40.3 | 13 | 15.3 | 75 | 24.9 |  |
|  | 7 | 13 | 17.3 | 5 | 7.2 | 26 | 36.1 | 11 | 12.9 | 55 | 18.3 |  |
| Hours/week exercise‡. | 0-5 hours | 11 | 14.7 | 7 | 10.1 | 3 | 4.2 | 19 | 22.4 | 40 | 13.3 |  |
|  | 6-10 hours | 36 | 48.0 | 34 | 49.3 | 23 | 31.9 | 30 | 35.3 | 123 | 40.9 |  |
|  | 11-15 hours | 13 | 17.3 | 23 | 33.3 | 26 | 36.1 | 27 | 31.8 | 89 | 29.6 |  |
|  | 16-20 hours | 11 | 14.7 | 3 | 4.3 | 16 | 22.2 | 6 | 7.1 | 36 | 12.0 |  |
|  | more than 20 hours | 4 | 5.3 | 2 | 2.9 | 4 | 5.6 | 3 | 3.5 | 13 | 4.3 |  |
| Reason for use | Medical | 24 | 32 | 30 | 43.5 | 26 | 36.1 | 19 | 22.4 | 99 | 32.9 |  |
|  | Recreational | 26 | 34.7 | 15 | 21.7 | 22 | 30.6 | 24 | 28.2 | 87 | 28.9 |  |
|  | Both | 25 | 33.3 | 24 | 34.8 | 24 | 33.3 | 42 | 49.4 | 115 | 38.2 |  |
| Cannabinoid types | THC | 13 | 17.3 | 10 | 14.5 | 20 | 27.8 | 18 | 21.2 | 61 | 20.3 |  |
|  | CBD | 21 | 28 | 31 | 44.9 | 25 | 34.7 | 24 | 28.2 | 101 | 33.6 |  |
|  | Both THC & CBD | 41 | 54.7 | 28 | 40.6 | 27 | 37.5 | 43 | 50.6 | 139 | 46.2 |  |
| Frequency cannabis use‡ | 3x weekly or less | 40 | 53.3 | 37 | 53.6 | 42 | 58.3 | 35 | 41.2 | 154 | 51.2 |  |
|  | 4x weekly-2x daily | 28 | 37.3 | 28 | 40.6 | 27 | 37.5 | 34 | 40 | 117 | 38.9 |  |
|  | More than 2x daily | 7 | 9.3 | 4 | 5.8 | 3 | 4.2 | 16 | 18.8 | 30 | 10 |  |
| Duration Use | < 3 years | 41 | 54.7 | 38 | 55.1 | 40 | 55.6 | 35 | 41.2 | 154 | 51.2 |  |
|  | More than 3 years | 34 | 45.3 | 31 | 44.9 | 32 | 44.4 | 50 | 58.8 | 147 | 48.8 |  |
| Do you take marijuana for pain? | No | 29 | 38.7 | 21 | 30.4 | 33 | 45.8 | 34 | 40 | 117 | 38.9 |  |
|  | Yes | 46 | 61.3 | 48 | 69.6 | 39 | 54.2 | 51 | 60 | 184 | 61.1 |  |
| Use within one-hour before exercise† | No | 53 | 70.7 | 54 | 78.3 | 61 | 84.7 | 54 | 63.5 | 222 | 73.8 |  |
|  | Yes | 22 | 29.3 | 15 | 21.7 | 11 | 15.3 | 31 | 36.5 | 79 | 26.2 |  |
| Use during exercise | No | 69 | 92 | 59 | 85.5 | 69 | 95.8 | 77 | 90.6 | 274 | 91 |  |
|  | Yes | 6 | 8 | 10 | 14.5 | 3 | 4.2 | 8 | 9.4 | 27 | 9 |  |
| Use within one-hour finishing exercise | No | 49 | 65.3 | 51 | 73.9 | 51 | 70.8 | 50 | 58.8 | 201 | 66.8 |  |
|  | Yes | 26 | 34.7 | 18 | 26.1 | 21 | 29.2 | 35 | 41.2 | 100 | 33.2 |  |

*Others refer to the following sports: swimming, winter sports, hiking, walking, climbing, yoga, trail running, and strength sports

Chi-square test: *p<0.05, †p<0.01, ‡p<0.001
