# Supplementary material for: Age related differences in cannabis use and subjective effects in a large population-based survey of adult athletes
Source: J Cannabis Res. 2019 Jul 29;1:7. doi: 10.1186/s42238-019-0006-9 (PMC7819305; doi:10.1186/s42238-019-0006-9)
Supplement: Supplementary file 2 — Table S1. Demographics and cannabis patterns of use by primary sport. (DOCX 27 kb) [file 42238_2019_6_MOESM2_ESM.docx]

Supplementary Table 2: Endorsement of reason for use within 1 hour before, during, and within one hour after exercise by age group*

| Time respect to exercise | Endorsement | Age | | | | | | | | | | | |
| --- | --- | --- | --- | --- | --- | --- | --- | --- | --- | --- | --- | --- | --- |
|  |  | 21 to 29  (n=26) | | 30 to 39  (n=44) | | 40 to 49  (n=34) | | 50 to 59  (n=18) | | 60 and older  (n=12) | | Total  (n=134) | |
|  |  | n | % | n | % | n | % | n | % | n | % | n | % |
| Within 1 hour before exercise | Pain management/relief | 5 | 19.2 | 15 | 34.1 | 13 | 38.2 | 10 | 55.6 | 4 | 33.3 | 47 | 35.1 |
|  | Improve focus, get in the flow | 10 | 38.5 | 28 | 63.6 | 14 | 41.2 | 6 | 33.3 | 4 | 33.3 | 62 | 46.3 |
|  | Improve activity enjoyment | 12 | 46.2 | 24 | 54.5 | 16 | 47.1 | 9 | 50.0 | 3 | 25.0 | 64 | 47.8 |
|  | Enhance performance | 6 | 23.1 | 15 | 34.1 | 14 | 41.2 | 4 | 22.2 | 3 | 25.0 | 42 | 31.3 |
|  | Relaxation | 7 | 26.9 | 8 | 18.2 | 11 | 32.4 | 7 | 38.9 | 2 | 16.7 | 35 | 26.1 |
|  | Increase energy | 7 | 26.9 | 16 | 36.4 | 16 | 47.1 | 3 | 16.7 | 3 | 25.0 | 45 | 33.6 |
|  | Aid in recovery | 1 | 3.8 | 3 | 6.8 | 7 | 20.6 | 2 | 11.1 | 0 | 0.0 | 13 | 9.7 |
|  | Aid in sleep | 2 | 7.7 | 5 | 11.4 | 9 | 26.5 | 3 | 16.7 | 1 | 8.3 | 20 | 14.9 |
| During exercise | Pain management/relief | 0 | 0.0 | 4 | 9.1 | 7 | 20.6 | 2 | 11.1 | 1 | 8.3 | 14 | 10.4 |
|  | Improve focus, get in the flow | 1 | 3.8 | 7 | 15.9 | 7 | 20.6 | 3 | 16.7 | 1 | 8.3 | 19 | 14.2 |
|  | Improve activity enjoyment | 3 | 11.5 | 12 | 27.3 | 7 | 20.6 | 3 | 16.7 | 3 | 25.0 | 28 | 20.9 |
|  | Enhance performance | 0 | 0.0 | 8 | 18.2 | 7 | 20.6 | 2 | 11.1 | 1 | 8.3 | 18 | 13.4 |
|  | Relaxation | 1 | 3.8 | 6 | 13.6 | 6 | 17.6 | 3 | 16.7 | 2 | 16.7 | 18 | 13.4 |
|  | Increase energy | 1 | 3.8 | 8 | 18.2 | 6 | 17.6 | 3 | 16.7 | 2 | 16.7 | 20 | 14.9 |
|  | Aid in recovery | 0 | 0.0 | 3 | 6.8 | 5 | 14.7 | 2 | 11.1 | 0 | 0.0 | 10 | 7.5 |
|  | Aid in sleep | 0 | 0.0 | 2 | 4.5 | 1 | 2.9 | 1 | 5.6 | 0 | 0.0 | 4 | 3.0 |
| Within 1 hour after exercise | Pain management/relief | 18 | 69.2 | 35 | 79.5 | 20 | 58.8 | 12 | 66.7 | 6 | 50.0 | 91 | 67.9 |
|  | Improve focus, get in the flow | 5 | 19.2 | 9 | 20.5 | 11 | 32.4 | 3 | 16.7 | 1 | 8.3 | 29 | 21.6 |
|  | Improve activity enjoyment | 6 | 23.1 | 12 | 27.3 | 10 | 29.4 | 2 | 11.1 | 3 | 25.0 | 33 | 24.6 |
|  | Enhance performance | 7 | 26.9 | 12 | 27.3 | 10 | 29.4 | 5 | 27.8 | 1 | 8.3 | 35 | 26.1 |
|  | Relaxation | 17 | 65.4 | 39 | 88.6 | 18 | 52.9 | 6 | 33.3 | 5 | 41.7 | 85 | 63.4 |
|  | Increase energy | 6 | 23.1 | 15 | 34.1 | 10 | 29.4 | 4 | 22.2 | 1 | 8.3 | 36 | 26.9 |
|  | Aid in recovery | 20 | 76.9 | 38 | 86.4 | 23 | 67.6 | 13 | 72.2 | 7 | 58.3 | 101 | 75.4 |
|  | Aid in sleep | 17 | 65.4 | 39 | 88.6 | 17 | 50.0 | 10 | 55.6 | 5 | 41.7 | 88 | 65.7 |

*The N’s for each age reflect those who endorsed cannabis use during that time period. Table data reflect those who endorsed the reported reason.
