# Supplementary material for: Age related differences in cannabis use and subjective effects in a large population-based survey of adult athletes
Source: J Cannabis Res. 2019 Jul 29;1:7. doi: 10.1186/s42238-019-0006-9 (PMC7819305; doi:10.1186/s42238-019-0006-9)
Supplement: Supplementary file 3 — Table S2. Endorsement of reason for use within one hour before, during, and within one hour after exercise by age group. (DOCX 19 kb) [file 42238_2019_6_MOESM3_ESM.docx]

## Demographics

#### What is your age?

( ) 21 to 29

( ) 30 to 39

( ) 40 to 49

( ) 50 to 59

( ) 60 to 69

( ) 70 or older

#### What is your gender?

( ) Male

( ) Female

( ) Other - Write In: _________________________________________________

( ) Prefer not to answer

#### What is your ethnicity?

( ) American Indian/Alaska Native

( ) Asian

( ) Black/African-American

( ) Hispanic/Latino

( ) Native Hawaiian or Other Pacific Islander

( ) White

( ) Prefer not to answer

( ) Other - Write In: _________________________________________________

#### What is your employment status?

( ) Full-time

( ) Part-time

( ) Unemployed

( ) Retired

( ) Student

( ) Other - Write In: _________________________________________________

#### What is your highest level of education?

( ) Less than high school

( ) Graduated high school

( ) Trade/technical school

( ) Some college, no degree

( ) Associate degree

( ) Bachelor's degree

( ) Advanced degree (Master's, Ph.D., M.D.)

#### Do you have pain?

( ) I have had pain for 3 or more months

( ) I have had pain for less than 3 months

( ) I don't have pain

## Athlete Profile

#### How many years have you been an athlete?

( ) 1-2 years

( ) 3-5 years

( ) 6-10 years

( ) 11-19 years

( ) 20 years or more

#### What is your primary sport/exercise?

( ) Swimming

( ) Running

( ) Cycling

( ) Triathlon

( ) Yoga/Pilates

( ) Climbing

( ) Spartan races

( ) Trail running

( ) Hiking

( ) Walking

( ) Strength training/gym

( ) Winter sports (skiing, snowboarding, snow shoeing)

( ) Martial arts/MMA

( ) Other - Write In: _________________________________________________

#### Over the past three months, on average, how many ****days per week**** do you exercise (include all sports that you participate in)?

( ) 1

( ) 2

( ) 3

( ) 4

( ) 5

( ) 6

( ) 7

#### Over the past three months, on average, how many ****hours per week**** do you exercise (include all sports that you participate in)?

( ) 0-5 hours

( ) 6-10 hours

( ) 11-15 hours

( ) 16-20 hours

( ) more than 20 hours

#### What is your athletic status?

( ) Professional

( ) Serious/competitive athlete (amateur)

( ) Frequent/fitness athlete

( ) Recreational athlete

( ) Other - Write In:

## Marijuana Use

#### In the past two weeks, have you used marijuana (including THC and/or CBD)?

( ) Yes

( ) No

#### Why do you currently NOT use marijuana? (check all that apply)

[ ] I don't need to use marijuana

[ ] I don't know enough about marijuana

[ ] Marijuana is not legal in my state

[ ] Marijuana is not legal at my job

[ ] I am scared to use marijuana

[ ] I am allergic to marijuana

[ ] I get sick from marijuana

[ ] I consider using marijuana doping

[ ] I am uncomfortable going to a dispensary

[ ] I can't afford it

[ ] Other - Write In: _________________________________________________

**Do you use marijuana medically, recreationally, or both?**

( ) Medically

( ) Recreationally

( ) Both medically & recreationally

**What do you primarily use THC, CBD, or both?**

( ) THC

( ) CBD

( ) Both THC & CBD

( ) Other - Write In: _________________________________________________

**How long have you been using marijuana?**

( ) 3 to <6 month

( ) 6 months to < 12 months

( ) 12 months to

( ) 3 years or more

( ) Not applicable

( ) Other - Write In: _________________________________________________

**How often do you use marijuana?**

( ) 1-3 times weekly

( ) 4-7 times weekly

( ) 1-2 times per day

( ) More than 2 times per day

( ) Other - Write In: _________________________________________________

( ) Not applicable

**How do you use marijuana?** (check all that apply)

[ ] Smoke in cigarette or pipe

[ ] Vaporizer

[ ] Spray

[ ] Capsule

[ ] Oil/tincture

[ ] Edible (cookie, candy, etc.)

[ ] Topical (cream, ointment, patch)

[ ] Other - Write In: _________________________________________________

[ ] Not applicable

#### Have you had any negative effects from marijuana use or contact? (check all that apply)

[ ] Respiratory (e.g. wheezing, coughing, itchy eyes, nasal symptoms)

[ ] Cardiovascular (e.g. increased heart rate, palpitations)

[ ] Gastrointestinal (e.g. nausea, vomiting, diarrhea)

[ ] Skin reaction (e.g. hives, rash)

[ ] Anxiety, paranoia, feeling uneasy

[ ] Worse athletic performance

[ ] Difficulty concentrating

[ ] Increased appetite

[ ] Other - Write In: _________________________________________________

[ ] No negative effects

**Have you had positive effects from marijuana use**? (check all that apply)

#### [ ] Increased energy

[ ] Helps with sleep

[ ] Euphoria

[ ] Calms me down

[ ] Decreased anxiety

[ ] Improved athletic performance

[ ] Less pain

[ ] Fewer muscle spasms

[ ] Decreased nausea

[ ] Other - Write In: _________________________________________________

[ ] No positive effects

**Do you take marijuana for pain?**

( ) Yes

( ) No

#### Do you use marijuana before, during and/or after exercise? (check all that apply)

[ ] Within one hour before starting exercise

[ ] During exercise

[ ] Within one hour finishing exercise

[ ] I don't use marijuana before, during, or after exericse

#### Why do you use marijuana before, during and/or after exercise? (check all that apply)

|  | **Within 1 hour before exercise** | **During exercise** | **Within 1 hour after exercise** |
| --- | --- | --- | --- |
| I don't use it during this time period | [ ] | [ ] | [ ] |
| Pain management/relief | [ ] | [ ] | [ ] |
| Improve focus, get in the flow | [ ] | [ ] | [ ] |
| Improve activity enjoyment | [ ] | [ ] | [ ] |
| Enhance performance | [ ] | [ ] | [ ] |
| Relaxation | [ ] | [ ] | [ ] |
| Increase energy | [ ] | [ ] | [ ] |
| Aid in recovery | [ ] | [ ] | [ ] |
| Aid in sleep | [ ] | [ ] | [ ] |
| Other | [ ] | [ ] | [ ] |
| Not applicable | [ ] | [ ] | [ ] |
